# Leptomeningeal metastasis from hepatocellular carcinoma with other unusual metastases: a case report

**DOI:** 10.1186/1471-2407-14-399

**Published:** 2014-06-03

**Authors:** Zhenyu Pan, Guozi Yang, Tingting Yuan, Xiaochuan Pang, Yongxiang Wang, Limei Qu, Lihua Dong

**Affiliations:** 1 Department of Radiotherapy, Norman Bethune First Hospital, Jilin University, 71 Xinmin Street, Changchun 130021, China; 2 Department of Radiology, Norman Bethune First Hospital, Jilin University, 71 Xinmin Street, Changchun 130021, China; 3 Department of Clinical Laboratory, Norman Bethune First Hospital, Jilin University, 71 Xinmin Street, Changchun 130021, China; 4 Department of Pathology, Norman Bethune First Hospital, Jilin University, 71 Xinmin Street, Changchun 130021, China

**Keywords:** Hepatocellular carcinoma, Metastasis, Leptomeningeal metastasis

## Abstract

**Background:**

Leptomeningeal metastasis, which results from metastasis of tumors to the arachnoid and pia mater, can lead to the dissemination of tumor cells throughout the subarachnoid space via the cerebral spinal fluid, and frequently with a poor prognosis. The primary tumor in adults is most often breast cancer, lung cancer, or melanoma. Although leptomeningeal metastasis due to cholangiocarcinoma has been reported, to the best of our knowledge there is no cytologically confirmed report of leptomeningeal metastasis from hepatocellular carcinoma.

**Case presentation:**

We herein report a case of leptomeningeal metastasis from hepatocellular carcinoma in a 53-year-old woman with concomitant systemic metastases to the lung, bone, brain, kidney, adrenal gland, subcutaneous tissues, and abdominal pelvis. The neurological symptoms of the patient were relieved after treatment with methotrexate intra-cerebral spinal fluid chemotherapy concurrent with whole brain radiotherapy.

**Conclusion:**

To our knowledge this is the first report of leptomeningeal metastasis from hepatocellular carcinoma confirmed by cytology. Treatment with methotrexate intra-cerebral spinal fluid chemotherapy concurrent with whole brain radiotherapy was effective.

## Background

Liver cancer is one of the most common malignancies and its highest prevalence is in China. The probability of distant metastasis of liver cancer is 30-50% [1], and the main metastatic routes are the portal vein, lymphatic channel, biliary tract, implantation, and the hepatic vein, among which the last is the most common. Metastatic foci often form hepatic arteriovenous fistulae via the hepatic venous system. This enables metastases of the tumor in the liver and other sites of the body [2]. Uncommon sites of metastases previously reported include the kidney and adrenal gland, retroperitoneum, pelvic lymph nodes, diaphragm, skin, brain, pleura, pancreas, gallbladder, stomach, mouth, bladder, and colon [1-5]. Although leptomeningeal metastasis due to cholangiocarcinoma has been reported [6], we did not find any relevant literature regarding confirmed leptomeningeal metastasis from hepatocellular carcinoma (HCC). Herein we report a rare case of leptomeningeal metastasis from HCC and review the relevant literature.

## Case presentation

A 53-year-old woman was admitted to our hospital due to fatigue, general malaise, and hiccups for one week. She had no history of chronic diseases or alcohol or tobacco. She had been working at a gas station for more than seven years.

**Figure 1:**
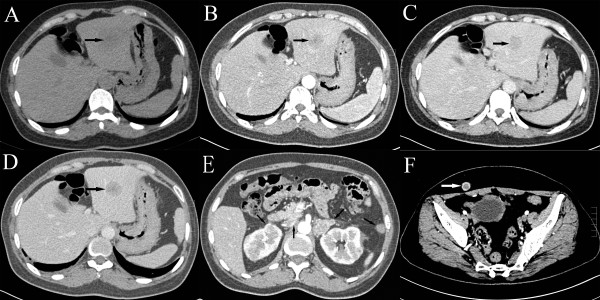
**Abdominal CT. A**-**D)** Plain scanning and triphasic contrast-enhanced scanning of lesions in the left liver lobe. **E)** Adrenal gland and kidney lesions. **F)** Lesions in the subcutaneous fat layer.

**Figure 2:**
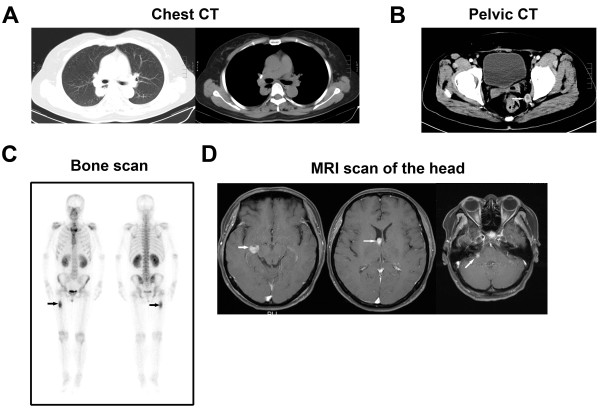
**Imaging findings. A)** Chest CT showed lesions in the inferior lobe of the right lung. **B)** Pelvic CT showed pelvic nodules. **C)** Bone scan showed increased radioactivity in the right femur. **D)** MRI scan of the head showed multiple lesions in the brain, with lesions in the right side of the cerebellopontine angle.

**Figure 3:**
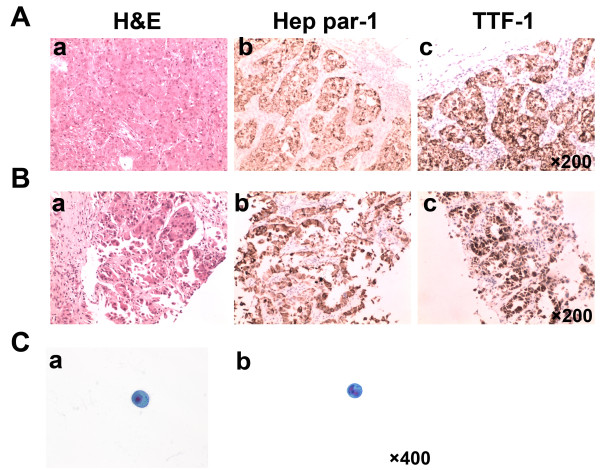
**Pathology slides of the resected specimen and CSF cytology slides. A)** Biopsy of subcutaneous tumor in the right shoulder. **B)** Biopsy of resected left liver lobe lesions. **C)** Cytological examination of CSF. Liquid-based technology, pap staining (400×).

On examination, painless subcutaneous nodules were found scattered throughout her body. Routine tests for hepatic and renal function, and blood and coagulation were normal, and hepatitis B, hepatitis C, syphilis, and human immunodeficiency virus tests were all negative. Tumor marker levels were: CA 125, 1438.0 U/mL; CA 153, 93.63 U/mL; CA 72–4, 300.0 U/mL; NSE, 13.4 ng/mL; CYFRA 21–1, 8.4 ng/mL; CA 199, 176.1 U/mL; AFP, 1.95 ng/mL; and CEA, 155.0 ng/mL.Computed tomography (CT) of the abdomen showed multiple lesions in the left lobe of the liver (Figure 1A-D), bilateral adrenal gland and kidney lesions (Figure 1E), multiple nodular lesions in the abdominal cavity (Figure 1E), and multiple nodules in the subcutaneous fat layer (Figure 1F). The chest CT scan showed lesions in the inferior lobe of the right lung and multiple subcutaneous nodular shadows (Figure 2A). The pelvic CT scan showed pelvic nodules and multiple subcutaneous nodular shadows (Figure 2B). The bone scan revealed increased radioactivity in the right femur (Figure 2C). The patient felt a headache on the third day of admission and the head magnetic resonance image showed multiple lesions in the brain and lesions in the right side of the cerebellopontine angle (Figure 2D).A subcutaneous tumor in the right shoulder was cut as biopsied, and the pathological examination showed metastatic cancer invasion (Figure 3A). Immunohistochemical analysis was positive for KI-67 (60%), CK20 (interspersed), CK, villin (focal), Hep Par-1, mammaglobin, CDX2, and TTF1; and negative for vimentin, GPC3, GCDFP-15, CK5/6, and napsin A. This confirmed the histological diagnosis of HCC. Histopathological examination of liver biopsy lesions revealed HCC (middle differentiation; Figure 3B). Immunohistochemical analysis was positive for CK7, CK20 (weak), CK18, CDX2 (weak), Hep Par-1, TTF1; and negative for GPC3, AFP, and napsin A. Hence, a diagnosis was made of HCC with metastases to the lung, bone, brain, kidney, adrenal gland, subcutaneous tissues, and abdominal pelvis. The Child-Pugh grade was A and the Karnofsky performance status score was 80 points.

The patient was treated with a systemic chemotherapy regimen: oxaliplatin 130 mg on day 1; leucovorin 300 mg on days 1–2; and tegafur 0.6 g on days 1–2.

The patient felt an aggravated headache three days after chemotherapy accompanied by hoarseness, dysphagia, and frequent vomiting, and the Karnofsky performance status score was 40 points. A lumbar puncture was performed and the results were: intracranial pressure 370 mm H_2_O, protein level 0.54 g/L, sugar level 3.69 mmol/L, and chlorine 96.9 mmol/L. Cytological examination of the cerebral spinal fluid (CSF) was performed via liquid-based technology (ThinPrep TCT2000) and the tumor cells were found through Papanicolaou staining (Figure 3C). Thus a diagnosis of leptomeningeal metastasis was made.

The patient was then given intrathecal chemotherapy (15 mg methotrexate and 5 mg dexamethasone, once a week) and concurrent whole brain and skull base radiotherapy (linear accelerator 6 MV photons, 2 Gy per day). Two weeks later, the symptoms were relieved except for dysphagia. However, jaundice appeared and the size and number of subcutaneous metastatic nodules increased. The liver function tests showed AST, 31 U/L; ALT, 53 U/L; ALP, 99 U/L; GGT, 53 U/L; TBIL, 76.6 μmol/L; DBIL, 33.2 μmol/L; and IBIL, 43.4 μmol/L. Tumor marker levels were: CA 125 > 5000 U/mL; CA 153 > 300 U/mL; CA 72–4 > 300 U/mL; NSE, 12.1 ng/mL; CYFRA21-1, 18.6 ng/mL; CA199, 976.4 U/mL; AFP, 3.28 ng/mL; and CEA, 311.7 ng/mL. Routine blood tests found hemoglobin 60 g/L and albumin 28.7 g/L. The Child-Pugh grade was C. After receiving radiotherapy (30 Gy/15 times) and intra-CSF chemotherapy (4 times), the patient refused radiotherapy and chemotherapy and only received supportive treatment. Her swallowing recovered one week later but relapsed after more than 40 days. The patient died due to tumor progression four months after diagnosis.

## Conclusion

Leptomeningeal metastasis is a rare complication in which metastatic tumor cells invade the leptomeninges (arachnoid and pia mater) of the meninx and arachnoid spaces. Leptomeningeal metastasis occurs in approximately 5% of people with cancer and is usually terminal. If left untreated, median survival is 4–6 weeks; if treated, median survival is 2–3 months [7]. Leptomeningeal metastasis is usually secondary to extracranial solid tumors such as breast cancer, lung cancer, and melanoma [8-10], while HCC commonly metastasizes to the lung, regional lymph nodes, peritoneum, adrenal glands, and brain [11]. To our knowledge, there have been no previous report in the literature describing leptomeningeal metastasis secondary to HCC, and this is the first confirmed by cytological examination.

In the present case, the diagnosis of HCC was made through pathological examination of two lesions (primary foci and metastases) combined with immunohistochemical results. Immunohistochemical examination of a subcutaneous tumor in the right shoulder tested positive for CK, Hep Par-1, CDX2, and TTF-1, and negative for vimentin, and napsin A. The liver biopsy lesions were positive for CK7, CK18, CDX-2, Hep Par-1, and TTF-1, confirming the histological diagnosis of HCC.

Most HCCs (70-90%) result from chronic liver disease and cirrhosis. The risk factors for HCC include virus infection, ingestion of toxin-contaminated food, and alcohol or autoimmune liver disease [12]. However, the patient in this report had no history of liver disease but had a long history of working at a gas station. We therefore speculate that tumorigenesis may be associated with long-term exposure of petroleum products.

Unlike other cancers that affect the central nervous system, the clinical signs and symptoms of leptomeningeal metastasis are highly variable because they affect the entire neuraxis. The clinical recognition of leptomeningeal metastasis is usually delayed due to ambiguous clinical signs and symptoms [7]. Although the diagnosis can be difficult, early diagnosis and leptomeningeal metastasis-related aggressive treatment can prevent irreversible neurologic deficits. The most common and definitive method for leptomeningeal metastasis diagnosis is the demonstration of malignant cells in the CSF, or spinal magnetic resonance imaging (MRI). CSF cytological analysis is the gold standard for identification [7]. In this case, cytological examination of the CSF was performed via liquid-based technology and tumor cells were found, confirming the diagnosis of leptomeningeal metastasis.

Brain metastases from HCC are extremely rare, accounting for only 0.2-2.2% of all metastases [5], but 40-70% of brain metastases can cause cerebral hemorrhage and are often associated with a poor prognosis. Previously reported brain metastases from HCC have complicated symptoms such as cranial nerve palsy, but a clear diagnosis of leptomeningeal metastasis using a CSF test was not performed [5,13,14]. The brain metastases in this patient were small and had no significant effect due to mass. In addition, we found lesions in the right side of the cerebellopontine angle, indicating cranial nerve injury. This case had no specific imaging of leptomeningeal metastasis such as thickness of the leptomeninx, but imaging was characteristic, including brain ventricle metastases and metastatic nodules near the pallium, which can assist the diagnosis. Furthermore, the patient had severe and diverse symptoms associated with the central nervous system, indicating leptomeningeal metastasis.

Leptomeningeal metastasis can be caused by hematogenous spread or invasion of brain metastases. If the metastatic lesions in the lateral cerebral ventricle lesion invade into the lateral ventricle ependymal, or the lesions in the right cerebral peduncle invade into the pia mater of the cisterna ambiens, the tumor cells may shed into the subarachnoid space and proliferate in the CSF, inducing leptomeningeal metastasis [7].

Leptomeningeal metastasis is a fatal complication of malignancies and difficult to treat; the median overall survival of treated patients is only 2 to 3months [15]. The goal of leptomeningeal metastasis-directed treatment is to stabilize the neurological status, improve quality of life, and prolong survival. Systemic and intra-CSF chemotherapy and site-specific radiotherapy are common treatment approaches [16]. In this report, the patient received concurrent radiotherapy and chemotherapy and achieved good effect without severe side effects. This may be because methotrexate is an antimetabolite drug and is cell cycle-specific, acting mainly on S phase. Since tumor cells in the S stage are resistant to radiotherapy, a combination of chemotherapy and radiotherapy can enhance the antitumor effect. Furthermore, methotrexate has a radio-sensitizing effect [17].

A randomized, multicenter, open-label study found that oxaliplatin plus a fluorouracil/leucovorin regimen had obvious benefits for treating advanced HCC with distant metastasis [18]. However, the patient in the present report was administered this regimen and incurred jaundice two weeks after the chemotherapy, consistent with hepatocellular jaundice, and the subcutaneous metastatic lesion significantly progressed with signs of anemia and hypoalbuminemia, indicating the tumor was uncontrolled.

In 30-40% of Chinese patients with HCC, alpha-fetoprotein is normal. Patients with high levels of alpha-fetoprotein frequently have a high grade of malignancy and poor prognosis [19]. For the patient of the present case, several tumor markers were significantly elevated but alpha-fetoprotein was normal. Imaging modalities indicated that liver malignancies comprised multiple nodular lesions, not typical of liver cancer.

This is a rare case in that the clinical features, biological behavior of the tumor, and response to treatment were quite different from most HCCs.

### Consent

Written informed consent was obtained from the next of kin of the patient for publication of this case report and any accompanying images. A copy of the written consent is available for review by the series editor of this journal.

## Abbreviations

HCC: Hepatocellular carcinoma; CT: Computed tomography; CSF: Cerebral spinal fluid.

## Competing interests

The authors declare that they have no competing interests.

## Authors’ contributions

All authors fulfill the authorship criteria because of their substantial contributions to the conception, design, analysis, and interpretation of the data. ZYP and GZY analyzed the data and drafted the manuscript. TTY, XCP, YXW and LMQ participated in data acquisition. LHD conceived the study, and participated in its design and in data acquisition. All authors read and approved the final manuscript.

## Pre-publication history

The pre-publication history for this paper can be accessed here:

http://www.biomedcentral.com/1471-2407/14/399/prepub
